# Leukocyte telomere length is associated with increased risk of endometriosis: a bidirectional two-sample Mendelian randomization study

**DOI:** 10.3389/fendo.2023.1272200

**Published:** 2023-11-16

**Authors:** Chenxue Bai, Zixiong Shen, Binxu Qiu, Songling Zhang

**Affiliations:** ^1^ Department of Obstetrics and Gynecology, The First Hospital of Jilin University, Changchun, China; ^2^ Department of Thoracic Surgery, The First Hospital of Jilin University, Changchun, China; ^3^ Department of Gastrointestinal Surgery, The First Hospital of Jilin University, Changchun, China

**Keywords:** endometriosis, telomere length, leukocyte telomere length, Mendelian randomization, endometrium

## Abstract

**Background:**

Endometriosis (EMs) is a common gynecological disorder. Observational studies on the relationship between leukocyte telomere length (LTL) and EMs have shown conflicting results. The purpose of this study was to evaluate the precise causal relationship between LTL and EMs using Mendelian randomization (MR) methodology.

**Methods:**

We employed MR to assess the causal relationship between LTL and EMs. Summary data from several large-scale genome-wide association studies (GWAS) were used for bidirectional two-sample MR analysis. Sensitivity analyses were conducted to ensure the robustness of our results. All analyses were also replicated in another completely independent EMs dataset.

**Results:**

Our MR analysis indicated that genetically predicted longer LTL increased the risk of EMs (IVW: discovery, OR=1.169, 95%CI: 1.059-1.290, p=0.002; validation, OR=1.302, 95%CI: 1.140-1.487, p=0.000), while EMs had no causal impact on LTL (IVW: discovery, OR=1.013, 95%CI: 1.000-1.027, p=0.056; IVW: validation, OR=1.005, 95%CI: 0.995-1.015, p=0.363). Causal estimates were supported by various calculation models (including MR-Egger, Weighted median, MR-PRESSO, and MR-RAPS). Heterogeneity and pleiotropy analyses also indicated robustness of the results.

**Conclusion:**

Our findings substantiate the idea that a genetically predicted longer LTL elevates the risk of EMs, with no influence of EMs on LTL risk. This research bolsters the causal link between LTL and EMs, overcoming the constraints of earlier observational studies. It implies that LTL may potentially function as a biomarker for EMs, opening up novel possibilities for EMs prevention and treatment.

## Introduction

Endometriosis (EMs) is a prevalent and intricate gynecological ailment characterized by the growth of endometrial-like tissue outside the uterine cavity, such as in the pelvic area, ovaries, and fallopian tubes (1). EMs poses a challenge for 5-10% of reproductive-aged women, often manifesting as pain, dysfertility, and discomfort during intercourse (2). The exact etiology of EMs remains unclear, with theories encompassing retrograde endometrial transplantation, embryonic developmental abnormalities, immune system aberrations, and genetic factors (3).

Telomeres are repetitive DNA sequences and associated proteins located at the ends of chromosomes (4). During cell division, a small portion of DNA is lost from the ends of chromosomes (4). Telomeres play a vital role in preserving the stability of crucial genes on chromosomes, and their length shortens with an increasing number of cell divisions (5). Critically short telomeres may lead cells to enter a state of aging or cease division, believed to be closely related to organismal aging (6). Conversely, longer telomeres play a significant role in maintaining cellular stability, delaying cell aging, sustaining stem cell function, and preventing cell apoptosis (6).

However, the relationship between TL and EMs has been a topic of debate. Studies suggest that TL may be associated with various gynecological diseases, including EMs (7). Additionally, the chronic inflammation associated with EMs may have an adverse effect on TL (8). One study (9) collected data from 877 women in New England (53 cases and 824 controls), revealing an association between shorter LTL and EMs (OR=2.56, 95%CI: 1.16-5.63; p=0.02). Conversely, another study (86 cases and 21 controls) found that EMs patients had higher peripheral blood LTL compared to the control group (OR=8.1, 95%CI: 1.28-51.57; p=0.0264) (10). A recent machine learning study (11) also identified telomere-related genes associated with EMs development, although their EMs sample was limited to 28 cases. These observational studies provide clinical evidence for the correlation between LTL and EMs, but unfortunately, their conclusions are not consistent. Moreover, their sample sizes are generally small, posing a risk of low statistical power. Therefore, conducting a large-scale study to explore the correlation between TL and EMs is necessary. Rahmioglu et al. (12) conducted a genome-wide association study (GWAS) meta-analysis of EMs, identifying 42 single nucleotide polymorphisms (SNPs) significantly associated with EMs. They comprehensively analyzed the genetic correlations between EMs and various pain and inflammatory diseases. However, they did not analyze the correlation between LTL and EMs. Nevertheless, their study provides data support for our research.

Mendelian randomization (MR) can be considered a natural randomized controlled trial (RCT) using genetic variations (typically SNPs) as instrumental variables (IV) for causal inference (13). MR is less susceptible to environmental influences because genetic variations are randomly allocated during meiosis and persist throughout a person’s lifetime (14). MR effectively circumvents confounding and reverse causation in observational studies and addresses the challenges of implementing RCTs (13, 14). By leveraging genetic information and large-scale GWAS, MR allows us to explore whether genetically predicted LTL contributes to the development of EMs and whether EMs, in turn, causally affect LTL.

To overcome the limitations of existing observational studies, we conducted a bidirectional two-sample MR study using large-scale GWAS data to reveal the causal relationship between LTL and EMs. Our study results, based on robust statistical methods and replication in an independent EMs dataset, provide compelling evidence. Our findings suggest that genetically predicted longer LTL increases the risk of EMs, while EMs do not causally impact LTL. These results not only enhance our understanding of the interplay between LTL and EMs but also emphasize the potential of LTL as a valuable biomarker for EMs. These insights could potentially alter our approaches to preventing and treating EMs, providing new pathways for therapeutic interventions and personalized care strategies.

## Method

### Study design

**Figure 1:**
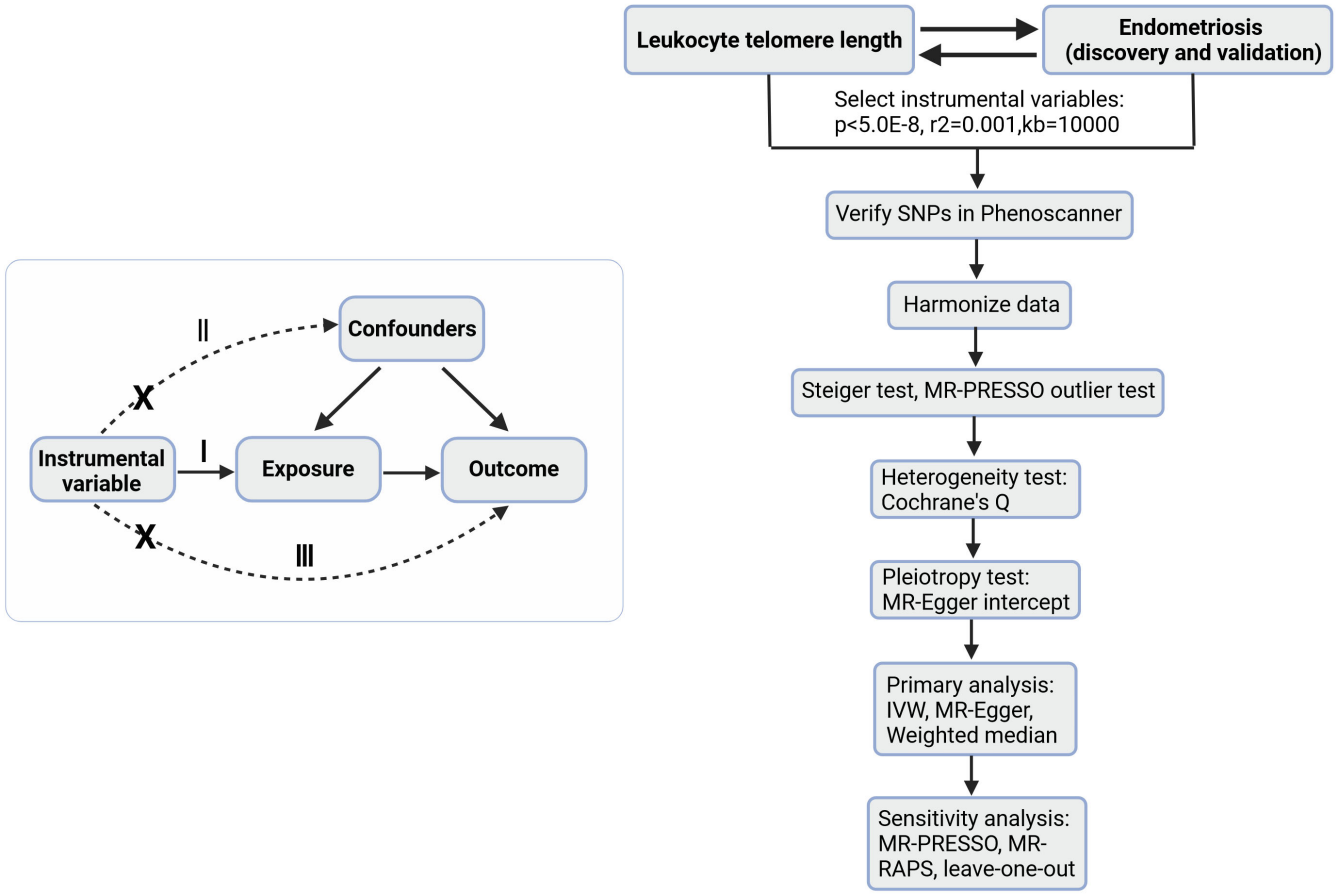
A brief description of the study. On the left is a bidirectional acyclic graph, on the right is the analysis flow of this study. I, assumption I; II, assumption II; III, assumption III; MR-Egger, MR-Egger regression; MR-PRESSO, Mendelian Randomization Pleiotropy RESidual Sum and Outlier test; MR-RAPS, Mendelian Randomization Robust Adjusted Profile Score.

**Figure 1** is a brief description of this study. This study is based on three basic assumptions of MR (15): I) The IV is associated with the exposure; II) The instrumental variables are independent of any known or unknown confounders that mediate the exposure to the outcome; III) The outcome is associated with the genetic instrument only through the effect of the exposure.

### Data Source

We utilized summary data from several large-scale GWAS studies in this research. Summary data for LTL were obtained from the UK Biobank (16), which comprised 472,174 individuals of European ancestry. LTL was defined as the average leukocyte telomere length measured using a multiplex quantitative polymerase chain reaction assay in a mixed leukocyte population, and then log-transformed to approximate a normal distribution (16). The GWAS data for EMs (discovery) was sourced from the meta-analysis conducted by Rahmioglu et al. (12) and included 21,779 European ancestry EMs cases and 449,087 European ancestry controls. The definition of EMs (discovery) encompassed a mix of surgically confirmed cases, medical records, and self-reported cases. For EMs (validation), the data were obtained from FinnGen (17) and comprised 15,088 cases of European ancestry and 107,564 controls of European ancestry. The definition of EMs (validation) was based on a mix of International Classification of Diseases 10 (ICD-10), ICD-9, and ICD-8 codes. Ethical approvals had been obtained for each GWAS dataset in their original studies, and our study solely utilized summary data, obviating the need for additional ethical approval. **Table 1** provides a brief overview of the GWAS data utilized in this study and how it was acquired.

**Table 1:** A brief description of each GWAS summary statistics.

Trait	Study/Consortium	Ancestry	Sample size	Cases definition	Data available
LTL	UK Biobank	European	472,174	The average leukocyte telomere length in a mixed white blood cell population measured using multiplex quantitative polymerase chain reaction technology.	“https://gwas.mrcieu.ac.uk/”; ID: “ieu-b-4879”
EMs(discovery)	The Women’s Health Study: From Adolescence to Adulthood; Crete dataset; DeCODE Genetics; The ENDOX study and Liverpool datasets; The ENDOX study part 2 and Liverpool and Edinburgh datasets; Leuven dataset; Lodz dataset; Melbourne dataset; Oxford Endometriosis Gene Study; Queensland Institute of Medical Research and Hunter; Community Study; University of California, San Francisco; Vanderbilt Biorepository; Danish Blood Donor Study; Generation Scotland: Scottish Family Health Study; The Estonian Biobank Cohort; Northern Finland Birth Cohort; Nurses’ Health Study II; UK Biobank dataset; QSkin Sun and Health Study; Twins UK; The Women’s Genome Health Study.	European	21,779 cases and 449,087 controls	Surgically confirmed (7593 cases); Medical records (797 cases); Self-reported (2791 cases); Mixed: Surgically confirmed + medical records (1716 cases); Mixed: Surgically confirmed + self-reported (2104 cases); Mixed: Medical records + self-reported (6778 cases).	“https://www.ebi.ac.uk/gwas”; ID: “GCST90269970”
EMs(validation)	FinnGen	European	15,088 cases and 107,564 controls	ICD-10-N80, ICD-9-617, ICD-8-6253	“https://www.finngen.fi/en”; ID: “N14_ENDOMETRIOSIS”

LTL, leukocyte telomere length; EMs, Endometriosis; ICD, International Classification of Diseases.

### Instrumental variable

We employed a significance threshold of p<5.0E-08 to identify SNPs significantly associated with both LTL and EMs. Stringent criteria were applied to remove linkage disequilibrium, with an aggregation window set at 10,000 kb and an r2 threshold set at 0.001. We calculated the F-statistic for each SNP and the overall F-statistic for the set of SNPs. The F-statistic for an individual SNP was determined using the following formula (18): $\text{F}=\frac{{\text{beta}}^{2}}{{\text{se}}^{2}}$ , where “beta” is the effect of the instrumental variable (IV) on the exposure, and “se” is the standard error of “beta.” The overall F-statistic was calculated using the following formula (18): $\text{F}=\frac{\text{N}-\text{K}-1}{\text{K}}\times \frac{{\text{R}}^{2}}{1-{\text{R}}^{2}}$ ${\text{R}}^{2}=2\times \text{eaf}\times (1-\text{eaf})\times {\text{beta}}^{2}$ , where “N” is the sample size for the exposure, “K” is the number of SNPs, “R^2^” is the proportion of exposure variance explained by SNPs, “eaf” is the effect allele frequency of the SNP, and “beta” is the effect of the SNP on the exposure. An F-statistic greater than 10 indicates a robust association between the SNP and the phenotype (19). We searched all SNPs in PhenoScanner to identify any SNPs related to potential confounders or outcomes. We then harmonized exposure and outcome data and excluded palindromic SNPs with moderate allele frequencies. Finally, we conducted an MR Steiger test to ensure the correct direction of causality (20) and removed SNPs that had a greater impact on the outcome than the exposure.

### Statistical analysis

We conducted a bidirectional two-sample MR analysis using LTL and Ems (discovery and validation). The primary analysis utilized the Inverse Variance Weighting (IVW) random-effects model, and we used MR-Egger regression and Weighted Median as validation methods. Heterogeneity was assessed using I^2^ and Cochran’s Q-value (21, 22), with I^2^>90% indicating reliable results (21). Assessment of the magnitude of pleiotropy was done by examining funnel plot symmetry and the difference in the intercept of MR-Egger regression from zero (23). Further sensitivity analysis was performed using Mendelian Randomization Pleiotropy RESidual Sum and Outlier test (MR-PRESSO) (24) and Mendelian Randomization Robust Adjusted Profile Score (MR-RAPS) (25) to address potential pleiotropy and weak instrument bias. MR-PRESSO was used to detect and correct for horizontal pleiotropy, with a distribution of MR-PRESSO set to 5000, and signifthreshold set to 0.05 in this study (24). MR-RAPS allowed for causal reevaluation after accounting for residual variance, effectively addressing horizontal pleiotropy and weak instrument bias (25). Finally, we conducted a leave-one-out analysis to identify individual SNPs that significantly affected the causal estimates.

MR results were presented in the form of odds ratios (OR) to establish the direction of causality (26). All analyses were performed using R software version 4.2.3 (https://www.r-project.org/). We used R packages such as “TwoSampleMR,” “MR-PRESSO,” and “mr.raps” for MR analysis, and data visualization was carried out using “TwoSampleMR” and “forestploter.” Instructions for using these packages can be found on GitHub (https://github.com). Lastly, we used the mRnd tool (27) to calculate the statistical power of the MR analysis (https://shiny.cnsgenomics.com/mRnd/).

## Results

### Instrumental variable

In the MR analysis with LTL as the exposure, initially, 154 SNPs were selected as Ivs for LTL. In PhenoScanner, these SNPs were not found to be associated with any potential confounders or outcomes. After removing palindromic SNPs and those missing in the outcome, 125 and 117 SNPs remained for the discovery and validation analyses, respectively. The Steiger test and MR-PRESSO outlier test did not identify any anomalous SNPs. Each SNP in this subset had an F-statistic greater than 10. The R^2^ for the 125 SNPs was 3.16%, with a total F-statistic of 123.33. For the 117 SNPs, the R^2^ was 2.93%, with a total F-statistic of 121.88.

In the MR analysis with Ems as the exposure, initially, 22 and 27 SNPs were selected as Ivs for Ems (discovery and validation, respectively). In PhenoScanner, these SNPs were not found to be associated with any potential confounders or outcomes. After removing palindromic SNPs and those missing in the outcome, 19 and 23 SNPs remained for the discovery and validation analyses. The Steiger test did not identify any anomalous SNPs. The MR-PRESSO outlier test detected 1 anomalous SNP in the Ems(validation) analysis, which was excluded from subsequent analysis. In the end, 19 and 22 SNPs were used for the discovery and validation analyses of Ems as the exposure. Each SNP in this subset had an F-statistic greater than 10. The R2 for the 19 SNPs was 6.41%, with a total F-statistic of 1696.01, and for the 22 SNPs, the R2 was 9.19%, with a total F-statistic of 564.23.

**Table S1**, **Table S2** and **Table S3** of supplementary tables provide the details of the initial selection of all SNPs in PhenoScanner in this study. **Table S4** contains information on SNPs that were not used in the final analysis. **Table S5** presents details on the SNPs used in the final analysis of this study.

### MR results and sensitivity analysis

#### Causal estimates

**Figure 2:**
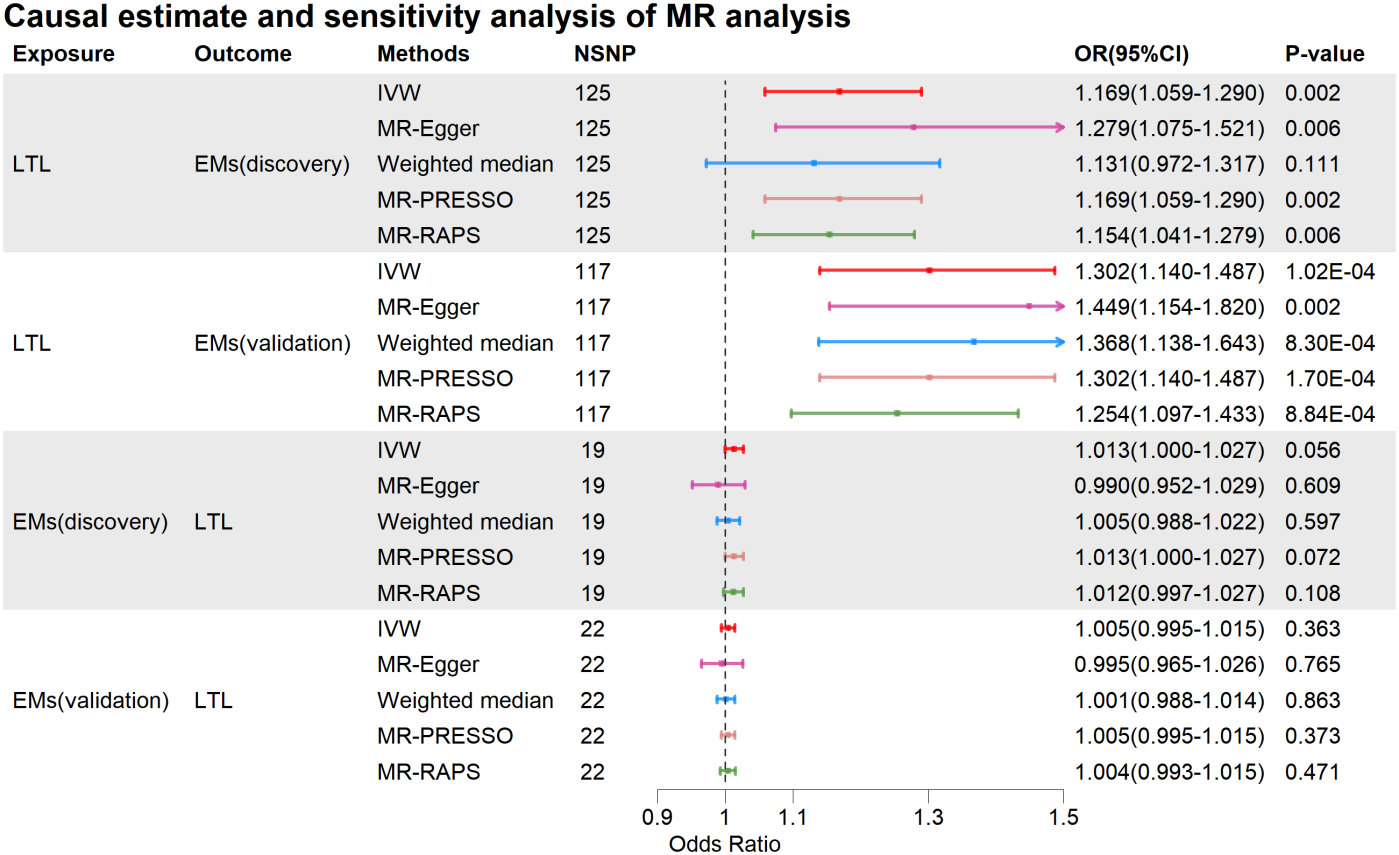
Causal estimate and sensitivity analysis of MR analysis. MR, Mendelian Randomization; NSNP, Number of SNPs; OR, odds ratio; LTL, leukocyte telomere length; Ems, Endometriosis; IVW, inverse variance weighting; MR-Egger, MR-Egger regression; MR-PRESSO, Mendelian Randomization Pleiotropy RESidual Sum and Outlier test; MR-RAPS, Mendelian Randomization Robust Adjusted Profile Score.

**Figure 3:**
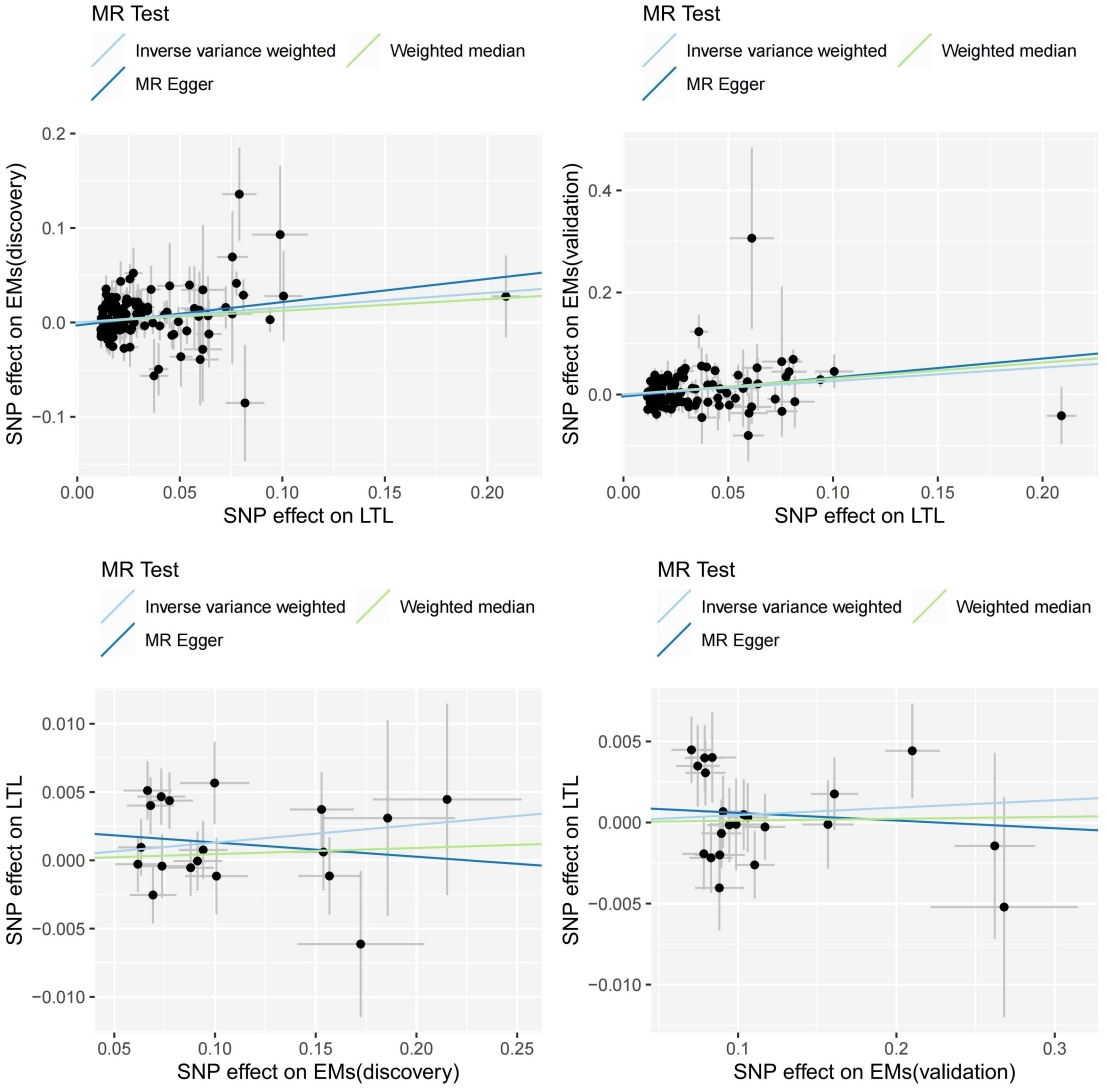
Scatter plots of MR analysis. MR, Mendelian Randomization; LTL, leukocyte telomere length; Ems, Endometriosis; SNP, single nucleotide polymorphism.

In summary, as depicted in **Figures 2**, **3**, our results suggest that genetically predicted longer LTL increases the risk of Ems, while Ems does not have a causal impact on LTL. The three primary methods, IVW, MR-Egger, and Weighted Median, consistently support the direction of causality (**Figure 3**). Our causal estimates were further validated in another entirely independent dataset of Ems. A series of sensitivity analyses further underline the robustness of our findings.

When LTL was considered as the exposure, the IVW analysis revealed a significant positive causal relationship between genetically predicted LTL and Ems (discovery, OR=1.169, 95%CI: 1.059-1.290, p=0.002; validation, OR=1.302, 95%CI: 1.140-1.487, p=1.02E-04). The IVW results in the discovery were supported by MR-Egger (OR=1.279, 95%CI: 1.075-1.521, p=0.006), and the Weighted Median maintained consistency with IVW in the direction of causal estimation (OR=1.131, 95%CI: 0.972-1.317, p=0.111). The IVW results in validation were supported by MR-Egger (OR=1.449, 95%CI: 1.154-1.820, p=0.002) and Weighted Median (OR=1.368, 95%CI: 1.138-1.643, p=8.30E-04). Furthermore, in both the discovery and validation, MR-PRESSO and MR-RAPS also supported the positive causal relationship between genetically predicted LTL and Ems. The mRnd tool calculated a statistical power of 100% for the MR analysis of LTL to Ems (discovery) and Ems (validation).

When Ems was used as the exposure, there was no evidence of a causal impact of Ems on LTL (IVW: discovery, OR=1.013, 95%CI: 1.000-1.027, p=0.056; validation, OR=1.005, 95%CI: 0.995-1.015, p=0.363). MR-Egger, Weighted Median, MR-PRESSO, and MR-RAPS also indicated that Ems did not causally affect LTL. More detailed MR causal estimation results are provided in **Supplementary Table S6**.

### Sensitivity Analysis

We assessed the heterogeneity and horizontal pleiotropy in our MR analysis. The results of heterogeneity and pleiotropy analyses in this study are presented in **Table 2**. **Figures S1** and **S2** depict funnel plots and leave-one-out analyses for Ems as both the outcome and exposure.

**Table 2:** Heterogeneity and pleiotropy of MR analysis.

Exposure	Outcome	Q	P-value	I^2^(%)	PRESSO-RSSobs	P-PRESSO	Egger-intercept	P-Egger
LTL	EMs(discovery)	148.22	0.068	99.20	151.09	0.062	-0.0031	0.221
LTL	EMs(validation)	202.03	0.000	99.18	205.70	0.000	-0.0038	0.259
EMs(discovery)	LTL	24.10	0.152	98.05	26.53	0.175	0.0024	0.231
EMs(validation)	LTL	24.54	0.268	98.35	26.54	0.288	0.0011	0.535

Q, Cochrane’s Q; I^2^, I squared; P-value, p-value of Q; PRESSO-RSSobs, RSSobs of Global Test in MR-PRESSO; P-PRESSO, p-value of PRESSO-RSSobs; Egger-intercept, intercept of MR-Egger; P-Egger, p-value of Egger-intercept; LTL, leukocyte telomere length; EMs, Endometriosis.

In MR analyses with LTL as exposure or outcome, some degree of heterogeneity was observed only in the MR analysis of LTL to Ems (validation). However, the MR-Egger intercept did not significantly differ from zero, and the leave-one-out analysis did not identify any single SNP with a significant impact on causal estimation results. This suggests that the presence of heterogeneity does not significantly affect the causal estimation results, and our results remain reliable. Additionally, in all other MR analyses, significant heterogeneity and horizontal pleiotropy were not observed, indicating high reliability and reproducibility of the results.

## Discussion

The exact cause of Ems remains uncertain. Current perspectives suggest it may be associated with embryonic development, degenerative physiological changes, immune factors, and genetic factors (3). Ems poses significant psychological and physiological burdens on women worldwide and their families. Over the past decade, numerous treatment approaches for Ems have emerged. Hormone therapy has shown promise (28), but it can lead to menstrual cycle changes, breast tenderness, mood swings, and headaches, and relapse is common upon discontinuation. Surgical intervention combined with medication is considered the gold standard for Ems treatment (29). However, surgery doesn’t address the root cause of Ems and can bring about tissue damage and a substantial economic burden that many patients find challenging to bear (29). Therefore, further research into the etiology of Ems can aid in disease prevention and early intervention, helping identify high-risk individuals. This is also of significant importance for drug development and adjusting treatment strategies to provide better medical care and psychological support for patients.

Rahmioglu et al. (12) conducted a comprehensive GWAS meta-analysis of Ems, identifying 42 significantly associated SNPs. They also analyzed the genetic correlations between Ems and various pain and inflammatory disorders, providing comprehensive insights into the associations between Ems and many diseases. However, there is currently no large-sample study on the correlation between LTL and Ems. Some previous observational studies suggested a link between LTL and Ems, but their conclusions were inconsistent. A case-control study included two large population studies to investigate the association between LTL and Ems (9). One group from New England, comprising 877 women (53 cases and 824 controls), showed a significant association with shorter LTL (OR=2.56, 95% CI: 1.16-5.63; p=0.02). The other group from the National Health and Nutrition Examination Survey, including 2268 women (151 cases and 2117 controls), indicated a similar but weaker association (OR=1.29, 95% CI: 0.85-1.96, p=0.22). Gleason et al. (30) reviewed data from the 1999-2002 National Health and Nutrition Examination Survey in the United States, finding that Ems patients had a shorter average LTL (-3.4, 95%CI: -7.3 to -0.3, p<0.05), and the LTL of Ems patients shortened by 1% per year. However, another observational study (86 cases and 21 controls) found that peripheral blood LTL in Ems patients was higher than in the control group (OR=8.1, 95%CI: 1.28-51.57; p=0.0264) (10). Some studies also investigated the association between TL in endometrial cells themselves and Ems. One study involving 29 cases and 27 controls measured the average TL of endometrial cells (31), and the results showed significantly longer TL in the Ems group (p = 0.005). Another study (32) measured replication characteristics and telomere length in endometrial cells of 38 Ems patients, indicating stronger replication status and longer average TL (p<0.05). These observational studies suggest that the causal direction between LTL and Ems remains unclear.

We addressed some limitations of observational studies in this research, providing new evidence to clarify the causal relationship between LTL and Ems. Our findings support a causal impact of LTL on Ems, rather than the other way around. Compared to previous observational studies, this research boasts several unique advantages. MR analysis is an effective epidemiological method that can overcome issues like confounding bias and reverse causality, which are challenging to resolve in some observational studies. This study also mitigated the limitations of smaller sample sizes in previous observational studies, offering more reliable causal inferences. Our study had a sufficiently large sample size, and we utilized a validation cohort, enhancing our statistical power.

Explaining how an increase in LTL raises the risk of Ems is indeed a challenge, and several potential mechanisms can elucidate this association. Firstly, previous study (33) has indicated a positive correlation between longer telomere length and enhanced cell proliferation and repair capabilities, along with the inhibition of apoptosis. Telomere length is also particularly closely associated with the division, growth, and maintenance of stem cells (34). Ems is believed to be linked to an excessive response of cyclic epithelial progenitor cells or stem cells related to endometrial regeneration after menstruation (35, 36). Therefore, we speculate that when endometrial tissue, carrying peripheral blood leukocytes, reach locations outside the uterine body, longer LTL may inhibit the apoptosis of ectopic endometrial cells and promote the cloning and differentiation of progenitor or stem cells. This could potentially facilitate the infiltration, survival, and unrestricted growth of endometrial cells in ectopic sites. Secondly, studies have shown a positive correlation between longer telomere length and estrogen levels (37), and estrogen plays a significant role in the pathological process of Ems (3). Excessive estrogen stimulation may lead to the growth and proliferation of ectopic endometrial tissues, thereby increasing the risk of Ems. Finally, LTL is influenced by genetic factors, resulting in variations in LTL between different individuals (38). Ems also exhibits certain features influenced by genetic factors (3), and some genetic factors may simultaneously affect both LTL and the development of Ems.

Research indicates that long-term chronic inflammation can lead to telomere shortening (39, 40), and Ems is a chronic inflammatory disease (3), providing a theoretical basis for how Ems may impact LTL. Previous observational studies have also observed telomere shortening in Ems patients (9, 30). However, our study did not find evidence of Ems causally affecting LTL. Nevertheless, negative results in MR studies cannot entirely exclude a causal relationship because genetic determinants of exposure may not represent the true exposure.

In summary, our study provides strong evidence regarding the association of increased Ems risk with longer LTL. These research findings may hold crucial clinical significance, particularly in the context of women’s health and patient care. They also provide valuable directions for future research. First, if LTL becomes an effective biomarker for Ems, it can aid in early diagnosis and intervention, ultimately reducing the severity of the disease and the suffering of patients. Second, for those already diagnosed with Ems, monitoring their peripheral blood LTL could assist in better disease management. Additionally, future research can explore how peripheral blood LTL influences the mechanisms behind Ems development. Finally, future studies can investigate whether adjusting LTL can reduce the risk of Ems or improve treatment outcomes. This could encompass interventions such as nutritional changes, lifestyle modifications, or drug therapies.

However, this study has limitations. Firstly, despite our efforts to mitigate pleiotropic bias using various methods, there is still a risk of potential pleiotropic bias, inherent to the limitations of the MR method itself (13). Secondly, all the summary data we used are from European populations, which limits the generalizability of the causal relationship to different ethnicities. Furthermore, our research focused on peripheral blood LTL and its relation to Ems, and the results may not represent the causal association of TL, particularly in endometrial cells themselves, with Ems. Lastly, we were unable to perform gender-stratified analyses due to the lack of appropriate data.

## Conclusion

In conclusion, our study strengthens the causal inference between LTL and Ems, supporting a positive causal impact of LTL on Ems, rather than Ems affecting LTL causally. This holds vital importance for deepening our understanding of the disease’s pathogenesis, offering potential avenues for Ems prevention and treatment. LTL may emerge as a potential biomarker for the disease, and future research can delve further into the exact role and impact mechanisms of LTL in Ems occurrence, including investigating interventions targeting LTL and related treatment methods.

## Data availability statement

Publicly available datasets were analyzed in this study. Leukocyte telomere length summary data can be obtained at “https://gwas.mrcieu.ac.uk/”, ID is “ieu-b-4879”. Endometriosis (discovery) summary data is available at “https://www.ebi.ac.uk/gwas” with ID “GCST90269970”. Endometriosis (validation) summary data are available at “https://www.finngen.fi/en/access_results”.

## Ethics statement

This study used summary data from genome-wide association studies and did not involve data from any individual individual. All data had received appropriate ethical approval in their original studies. No additional ethical approval was required to conduct this study.

## Author contributions

CB: Formal Analysis, Investigation, Methodology, Project administration, Resources, Software, Supervision, Validation, Writing – original draft, Writing – review & editing. ZS: Conceptualization, Data curation, Formal Analysis, Investigation, Methodology, Project administration, Software, Validation, Writing – original draft, Writing – review & editing. BQ: Data curation, Formal Analysis, Investigation, Methodology, Validation, Writing – original draft, Writing – review & editing. SZ: Data curation, Methodology, Resources, Supervision, Validation, Visualization, Writing – original draft, Writing – review & editing.
