# Supplementary figures and images for: Leukocyte telomere length is associated with increased risk of endometriosis: a bidirectional two-sample Mendelian randomization study
Source: Front Endocrinol (Lausanne). 2023 Nov 16;14:1272200. doi: 10.3389/fendo.2023.1272200 (PMC10687575; doi:10.3389/fendo.2023.1272200)

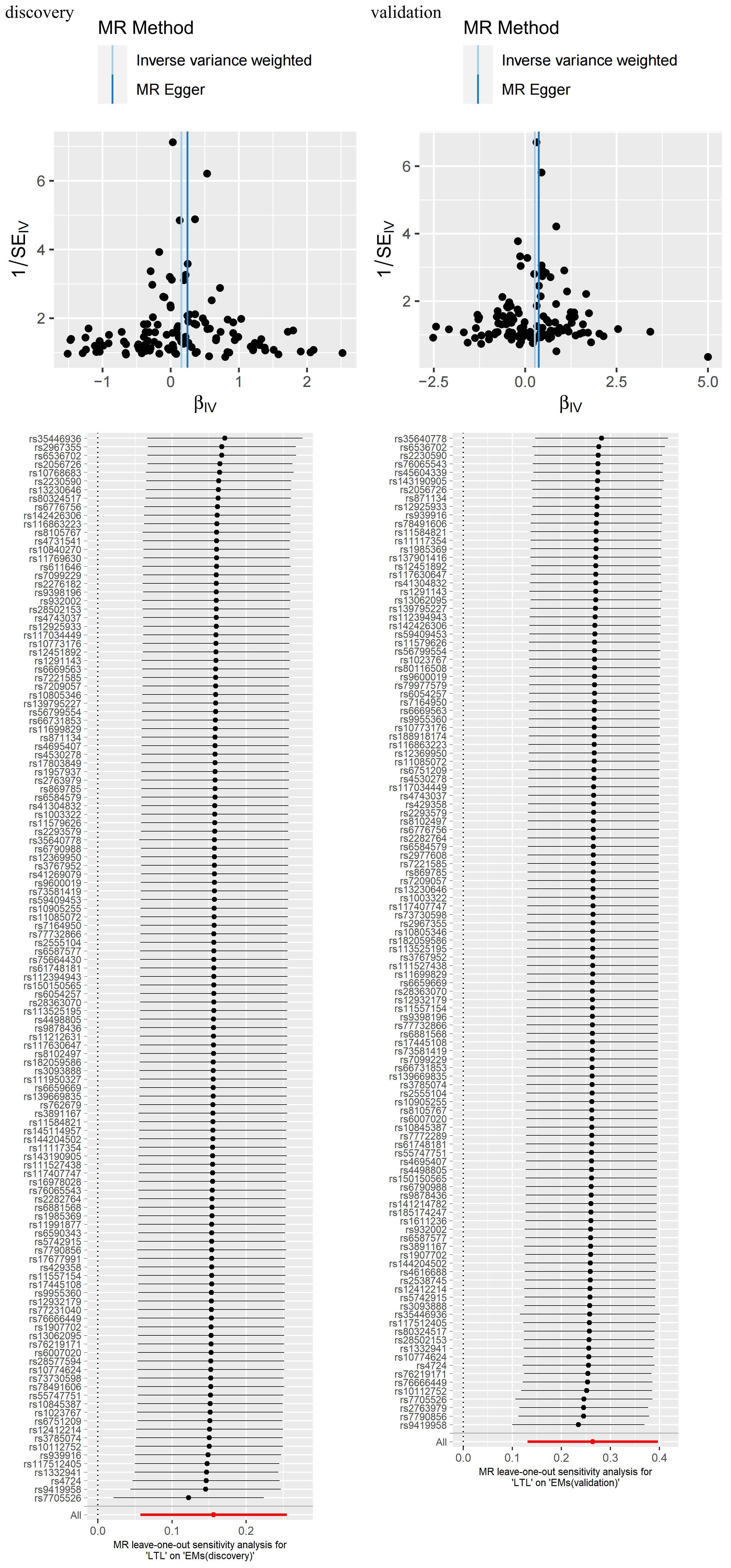

Supplement: Supplementary file 1 [file Image_1.jpg]

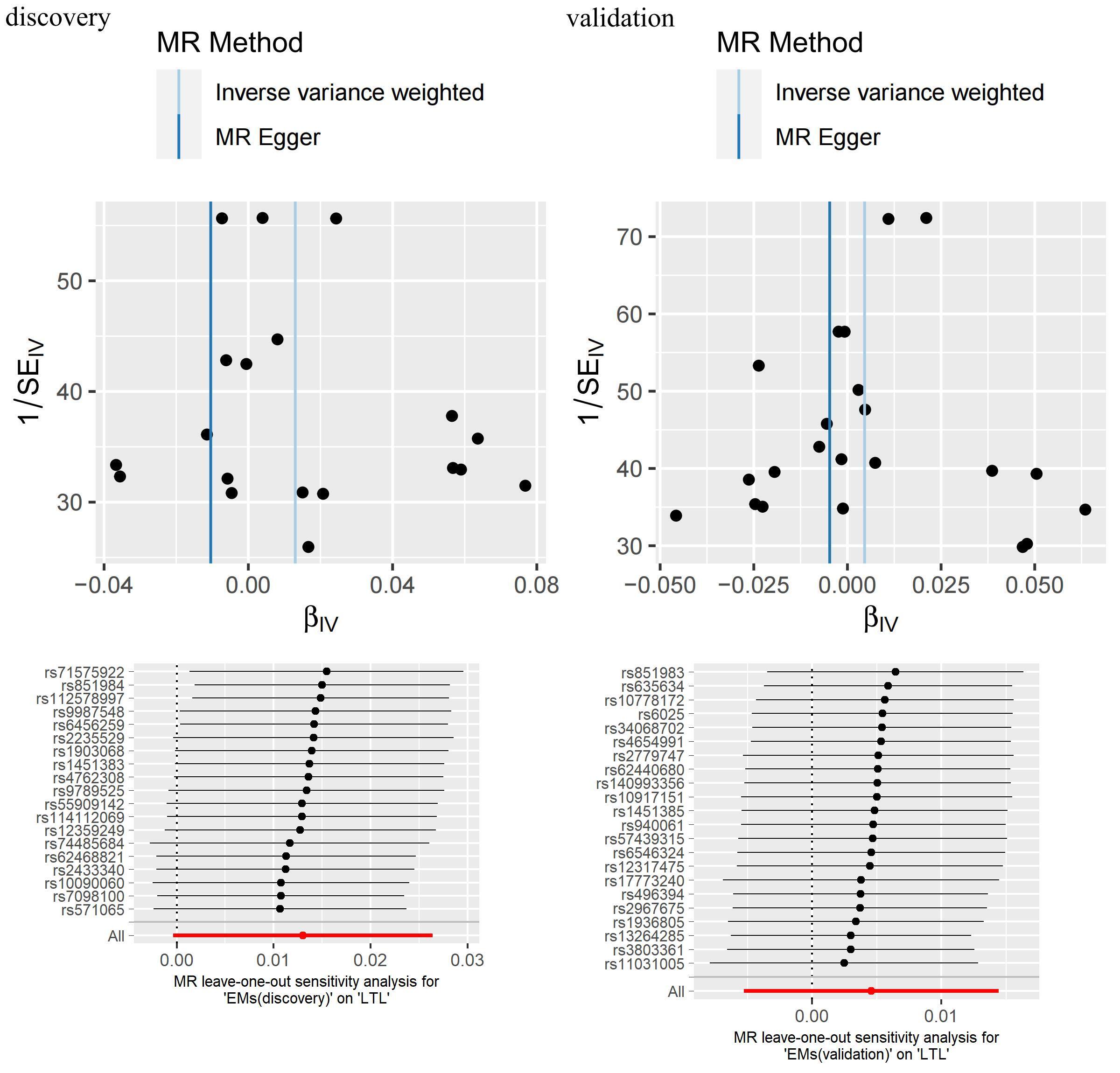

Supplement: Supplementary file 2 [file Image_2.jpg]
